# Should We Change the Target of Therapy in Pulmonary Hypertension?

**DOI:** 10.3390/life13051202

**Published:** 2023-05-17

**Authors:** Panagiotis Karyofyllis, Eftychia Demerouti, Pavlos Habibis, Styliani Apostolopoulou, Eleftheria-Garyfallia Tsetika, Dimitrios Tsiapras

**Affiliations:** 1 Cardiology Department, Onassis Cardiac Surgery Center, 17674 Athens, Greece; 2 School of Medicine, University of Thessaly, 41221 Larissa, Greece

**Keywords:** pulmonary arterial hypertension, mean pulmonary arterial pressure, epoprostenol, balloon pulmonary angioplasty, chronic thromboembolic pulmonary hypertension, survival, right ventricle

## Abstract

Despite the evolution of drug therapy in pulmonary arterial hypertension and the more aggressive treatment approach according to the guidelines, patients continue to have unacceptable mortality rates. Furthermore, specific drug therapy alone in chronic thromboembolic pulmonary hypertension also does not seem to have any beneficial impact on survival. As the function of the right ventricle (RV) determines the prognosis of patients with pulmonary hypertension, the treatment strategy should focus on modifying factors involved in RV dysfunction. Although some previous reports demonstrated that the survival of patients with pulmonary hypertension was associated with mPAP, nevertheless, mPAP is still not considered as a target of therapy. There are many examples of effective mPAP lowering with early and aggressive drug therapy in pulmonary arterial hypertension, or with interventions in chronic thromboembolic pulmonary hypertension. This effective mPAP reduction can lead to reverse RV remodeling, and thus, improvement in survival. In this article, the importance of mPAP lowering is stated, as well as why the change of our current strategy and considering mPAP reduction as the target of therapy could make pulmonary hypertension a chronic but not fatal disease.

## 1. Introduction

Pulmonary arterial hypertension (PAH) is a chronic and progressive disease leading to right heart failure and ultimately death in a rather short period of time if left untreated. Another form of severe precapillary pulmonary hypertension (PH), a progressive pulmonary vascular disorder also resulting in right heart failure and death, is chronic thromboembolic pulmonary hypertension (CTEPH), which is considered a complication of pulmonary embolism. These two forms of precapillary PH may share similar pathological changes in the distal pulmonary vasculature, including the intimal thickening and remodeling of pulmonary resistance vessels, eccentric intimal fibrosis, intimal fibromuscular proliferation, and plexiform lesions [1]. Besides these similarities, the main difference is that in CTEPH, there is a significant mechanical component amenable to interventional treatment.

While the first classification of PH was proposed in Geneva [2] in 1973, designating only two groups, idiopathic and secondary PH, it took almost two decades since then for the community to learn the natural history of primary PH, as it emerged from the publication of the results of the NIH registry [3] in the era of non-specific treatment. This registry revealed the devastating nature of the disease, as the median survival for subjects diagnosed with idiopathic pulmonary arterial hypertension was approximately 2.8 years. Intravenous epoprostenol was introduced in 1995 as the first approved disease-specific targeted medical therapy for PAH. Since then, more than ten specific drugs administered by all four routes of administration (i.v., s.c., oral, and inhaled) have now become available. However, what did we gain in the era of specific PAH treatments? Despite the improvement in short-term survival using new effective therapies, PAH remains an incurable disease with an unacceptable median survival of 7 years [4]. This fact could raise the question of whether these drugs are effective enough, or if we do not use them appropriately.

## 2. Risk Stratification and Treatment Goal in Pulmonary Hypertension

The 2022 ESC/ERS PH guidelines [5] have recommended a baseline patient risk assessment using a multi-dimensional stratification according only to modifiable clinical, functional, biochemical, imaging, and hemodynamic variables with known prognostic significance, categorizing patients as low, intermediate, or high risk, depending on the risk of death in one year. A four-strata risk tool is additionally recommended for the follow-up assessment; this tool ignores the functional parameters of the right ventricle (RV), but the target of therapy remains the same: the achievement of low-risk status. However, a low risk does not mean no risk, and given the progressive nature of the disease, if the main factor that is responsible for the altered structure and function of the RV is not modified, then the disease will still be considered fatal.

Many etiologies are considered responsible for the evolution of PH, but they all share the same underlying mechanism of elevated pulmonary arterial pressure resulting in an increase in right ventricular afterload. This increase in afterload results in a spectrum of mechanical and biochemical changes, both adaptive and maladaptive, that may lead to the syndrome of right heart failure.

Right heart failure is the predominant cause of death in PAH patients [6], and the survival of PAH patients is strongly related to the avoidance of right heart failure, a consistent finding among large cohorts and smaller studies [7]. There is significant variability in the response of the right heart to PAH treatment, and the identification of patient-specific factors, allowing a more personalized approach to therapy, is needed. However, a characteristic of survival risk prediction models for PAH is the absence of an RV function assessment, at least at follow-up. Hypothetically, if normal RV function were the only goal during the follow-up of PAH patients, their survival would be better. So, should we change the target of PH treatment?

## 3. The Right Ventricle: The Key Factor for Survival

The RV is physiologically coupled to a high-compliance, low-resistance pulmonary circulation as a single cardiopulmonary unit, and low pressure is sufficient to pump the blood to the lungs for oxygenation. The RV seems to adapt better to changes in volume rather than pressure, and thus, afterload is a primary determinant of normal RV function; minor increases in afterload can cause significant decreases in RV stroke volume and, therefore, right ventricular ejection fraction [8].

The increase in pulmonary vascular resistance (PVR) results in an increase in RV wall stress, which is a crucial driver of RV changes from pressure overload. As pulmonary artery (PA) pressure rises, the pulmonary circulation becomes a high-pressure, high-resistance system, like systemic circulation, which adds excessive load to the contracting RV, altering the RV-PA coupling. As vascular pathology in pulmonary hypertension subsets leads to a chronic increase in afterload, the RV has to adapt by hypertrophy and a compensatory increase in contractility to keep the RV-PA coupling constant. Especially in precapillary forms of PH, the vascular disease progressively deteriorates, intensifying the afterload mismatch; the RV dilates in an effort to maintain stroke volume by virtue of the Frank–Starling law, and its contractility becomes insufficient to support adequate function, leading to RV-PA uncoupling.

End-systolic elastance is a load-independent measure of contractility, whereas vascular load (RV afterload) is determined by the arterial elastance. The ratio between RV contractility (end-systolic elastance) and RV afterload (arterial elastance) describes the concept of RV–PA coupling. In physiologic conditions, the Ees/Ea ratio is between 1.5 and 2, allowing the RV flow output to have a minimal energy cost and optimal RV-PA coupling [9]. This ratio could approximately be estimated from ordinary hemodynamic measurements by the mathematical assumption of maximum RV pressure/mean pulmonary arterial pressure (mPAP)-1 [10]. Thus, in PH cases without intrinsic myocardial damage, which would have led to irreversible RV dysfunction, simply by reducing mPAP, RV-PA coupling could move toward normal conditions, and mPAP lowering would result in the recovery of RV function. As long-standing PH has been shown to result in disrupted matrix turnover and RV fibrosis [11], which could be considered irreversible damage, the effort for a substantial reduction in mPAP and PVR would be more effective in the early course of the disease, as the damage in pulmonary vasculature would be still reversible. In cases with severe intrinsic myocardial damage, the systolic function of the RV could deteriorate to the point at which it is unable to generate adequate PAP and flow. Thus, the reduction in mPAP in such cases is a sign of deterioration and should not be considered as the target of therapy without the concomitant improvement of RV function.

Does long-standing PH cause truly irreversible damage in the RV? The importance of mPAP reduction is also evident from the fact that even in the very late stage of the disease, RV function could be restored if, hypothetically, mPAP could be normalized. The best proof for this consideration comes from transplanted patients, where almost any RV recovers within a few weeks after lung transplantation, regardless of the degree of pre-transplant dilatation and dysfunction [12].

RV dysfunction in PH is the major determinant of mortality; improved or normalized RV function after our therapeutic interventions in PH could lead to effectively better survival. The devastating forms of PH, especially if left untreated, are PAH and CTEPH. Early and mainly effective therapeutic interventions in these two forms of PH have been proven to confer a beneficial impact on survival by reversing the remodeling of the RV, but how feasible is the normalization or near normalization of mPAP or PVR in such PH patients?

## 4. Pharmacotherapy in PAH

The management of PAH has substantially improved in the last decade. An expanded understanding of PAH pathophysiology led to advances in targeted drug therapy, targeting the nitric oxide (NO), endothelin-1, and prostacyclin pathways (prostaglandin I2) (PGI2). There are currently several drugs available targeting these pathways, including phosphodiesterase-5 inhibitors (PDE5is) such as sildenafil and tadalafil and the soluble guanylate cyclase (sGC) stimulator (Riociguat); endothelin receptor antagonists (ERAs) such as ambrisentan, bosentan, and macitentan; and Prostacyclin class agents. Prostacyclin agents are administered intravenously, subcutaneously, and by inhalation. Recently, oral selective IP prostacyclin receptor agonists, Selexipag and Ralinepag, are available for non-high-risk patients.

ERAs and PDE5is are routinely used as an initial combination therapy for the majority of PAH patients. Parenteral prostanoids are predominantly administered to high-risk patients. The sGC stimulator Riociguat and the oral prostacyclin receptor agonist Selexipag have been recently used during patients’ follow-up when low-risk status is not achieved. Initial or sequential combination therapy has become a widely adopted treatment strategy in PAH, simultaneously targeting more than one of the signaling pathways implicated in disease progression. The latest guidelines highlight the early administration of parenteral prostanoids for intermediate–high-risk patients during their follow-up [5], as these drugs are proven to improve survival in PAH via RV function improvement and/or normalization.

As PAH remains an incurable condition with a high mortality rate despite the use of PAH-specific drugs targeting the three pathophysiological pathways of the disease, novel agents are currently in phase 3 development, such as sotatercept. Sotatercept acts as a ligand trap for members of the transforming growth factor (TGF)-β superfamily, thus restoring balance between growth-promoting and growth-inhibiting pathways.

## 5. The Effective RV Afterload Reduction in PAH

The significance of lowering PAP in guiding pharmacotherapy has not been widely described so far. In patients with PAH who are responders to vasodilator challenge, high-dosage calcium channel blockers can achieve an impressive reduction in mPAP (239%) and PVR (250%) with the best long-term survival [13] and, consequently, a near normalization of RV function [14]. Despite the proven excellent long-term survival of vasoreactive patients whose mPAP can be sufficiently decreased [15], mPAP is not currently recognized as a target for PAH treatment. Indeed, these patients are considered to have a different phenotype from the non-responders, but the aggressive lowering of mPAP could result in their favorable prognosis.

As combination therapy targets multiple pathophysiological pathways, it is considered the standard of care in PAH, and initial oral combination therapy is recommended for non-high-risk patients. Studies with oral combination therapy have shown a statistically significant reduction in mPAP and PVR compared to oral monotherapy, but this reduction is arithmetically modest. In a retrospective analysis of the real-world clinical data of 97 patients with newly diagnosed PAH, the initial dual oral combination treatment with an ERA plus a PDE-5 inhibitor resulted in a mean mPAP reduction of <10 mmHg [16]. In another retrospective study with incident PAH patients, upfront oral combination therapy also resulted in a mean change in mPAP of −11 mmHg after 1 year of follow-up, with a concomitant improvement in right ventricular ejection fraction, volumes, and mass [17]. Even in scleroderma-associated PAH, with the known dismal response to therapy, the combination of ambrisentan and tadalafil showed a mean reduction in mPAP of 12 mmHg [18]. Ralinepag is a novel oral selective, non-prostanoid prostacyclin receptor agonist targeting the prostacyclin pathway, and its use in double or triple combination therapy with other oral drugs resulted in a 6.1 mmHg mean reduction in mPAP [19].

Similar findings were provided by the OPTIMA study, where the combination of macitentan and tadalafil led to a mean reduction in mPAP of 7.8 mmHg [20]. Finally, in the TRITON study, a similar modest reduction in mPAP of <13 mmHg was observed irrespective of whether the patients were on dual or triple oral combination therapy [21].

Epoprostenol was the first drug approved for PAH and may still be the most effective in lowering mPAP and PVR, and consequently improving survival. Epoprostenol is known to be the most potent drug among all the PAH-targeted drugs. In a pilot study with upfront triple combination therapy including epoprostenol, mPAP decreased by 32% and PVR by 71%. Furthermore, the survival rate after 3 years of follow-up was 100%, even though the dose of epoprostenol was low (<20 ng/kg/min) [22]. In a study [23] conducted by Ogawa et al., intravenous epoprostenol was highly prescribed, as >78% of patients were on epoprostenol therapy. The mean survival time from diagnosis was 14.9 ± 0.8 years (95% CI, 13.4–16.4 years), and hemodynamic parameters improved significantly, as treatment decreased mPAP by >37% and PVR by >61%. The significant improvement in survival for patients treated with intravenous epoprostenol is attributed to PVR and mPAP reduction, and dosage seems to have a crucial role in the patient’s hemodynamic improvement. An interesting study from Japan reported that IPAH patients treated with high-dose epoprostenol showed marked hemodynamic improvement [24]. An average dose of 107 ± 40 ng/kg/min epoprostenol was used, and subsequently, mPAP decreased from 66 ± 16 to 47 ± 12 mmHg, and, more importantly, the survival rate was 100% for a period of 3.7 years. A single-center retrospective study [25] including patients with idiopathic/familial PAH reported an average epoprostenol dose of 80 ng/kg/min, with a substantial reduction in mPAP and PVR, by 44% and 67%, respectively, and a 5-year survival rate of 96%, which was much better than the reported 5-year survival rate of 65% in the REVEAL registry [4]. However, the most impressive and worth mentioning finding in this study was that for patients in whom a decrease in mPAP <42.5 mmHg was achieved, the 10-year survival rate was 100%. Badagliacca et al. analyzed the “normalization” of mPAP to <25 mmHg with treatments based on ERAs, PDE5is, and parenteral prostanoids in 267 consecutive patients with PAH [26]. Over an average of 58 months of follow-up, the authors concluded that a reduction to an mPAP <35 mmHg may be a meaningful treatment goal, as this value was defined as the best cut-off for survival prediction. In addition to epoprostenol, another parenteral prostanoid, subcutaneous treprostinil showed favorable hemodynamic effects in upfront combination with ambrisentan and tadalafil [27]. Furthermore, at a median follow-up of 2 years, all patients remained alive.

It is crucial to act as soon as possible with the goal of achieving near-normal hemodynamics before lesions to the pulmonary arterial tree become irreversible. This statement is supported by studies from Japan, where the early use of epoprostenol is common practice. Tokunaga et al. [28] reported that a rapid up-titration of epoprostenol soon after initiation was associated with a continuous reduction in mPAP and a 9.5-year survival rate of 100%. In contrast, the slow increase group did not demonstrate such reductions in mPAP despite the similar final epoprostenol dose, and also, the survival rate was worse (64%). Rapid and sufficient vasodilation caused by increasing doses of epoprostenol would diminish the deleterious impact of high PAP, which would promote pulmonary remodeling and right ventricular pressure overload [29]. The beneficial effects of early intensive PAH treatment, with the feasibility of effective mPAP reduction and the importance of considering mPAP as a target of therapy, were demonstrated in the retrospective study of Sugiyama et al. [30]. In this study, the therapeutic goal was the achievement of an mPAP < 40 mmHg in the treatment of naïve patients, with the immediate escalation of therapy within 3 months after treatment initiation. As a result, 86% of the PAH patients achieved an mPAP < 40 mmHg with an intensive treatment algorithm aiming to lower mPAP. Notably, mPAP could be lowered to <25 mmHg in 47% of patients. The survival rate of the patients who achieved the therapeutic goal (3- and 5-year survival rate: 97.3%) was significantly better than that of the patients who did not reach the therapeutic goal (3-year survival rate: 50.0%, *p* < 0.05).

Table 1 summarizes the studies with significant reductions in mPAP, the survival rate observed, and the underlying treatment strategy including parenteral prostanoids.

All the aforementioned observations show the magnitude of mPAP lowering for improving the outcome of PAH patients. A pulmonary artery pressure-directed multi-drug approach for PAH may reverse right heart remodeling and limit progression, or even reverse pulmonary vascular disease [27]. The majority of PH specialists believe that once elevated, it is not feasible for mPAP to be sufficiently decreased by specific treatment, and this consideration may be based on the results of the available studies. However, all the above-mentioned data suggest that a reduction in mPAP is feasible, but for a reduction that will be reflected in an improvement in the function of the RV and, by extension, in survival, we must change strategy and consider the effective lowering of mPAP as a target of therapy. So, we must change the way we are thinking and use early combination therapy including prostanoids, considering mPAP as a target of therapy. As this strategy seems feasible, a near-normal hemodynamic profile within a short time period should be the goal of treatment. Still, if this goal is not achieved, epoprostenol should be considered a necessary drug to be added to the oral combination drug therapy [31].

## 6. The Effective RV Afterload Reduction in CTEPH

CTEPH is a vascular disorder characterized mainly by a mechanical component due to the obstructive lesions in the pulmonary artery tree caused by organized fibrotic thromboembolic material and variable small vessel disease due to the remodeling of small muscular pulmonary arteries. In CTEPH, the level of mPAP, among other parameters, is a crucial determinant of survival [32]. It is obvious that no one of the available specific PH drugs can provide relief from the mechanical component of the disease, and this is the reason for their ineffectiveness in substantially lowering mPAP or effectively improving survival.

In three randomized placebo-controlled trials with bosentan [33], macitentan [34], and riociguat [35], the treatment effect in reducing mPAP was meaningless, −2.5, −1.9, and −4 mmHg, respectively, even though the statistics show it to be statistically significant for riociguat. A similar treatment effect (−3.4 mmHg) was also achieved with subcutaneous treprostinil in another randomized trial [36]. These reductions mean that in the population of the aforementioned trials, mPAP remained ≥40 mmHg, given the baseline values, which correlates to a 5-year survival rate of <40% according to Riedel et al.’s observations [32]. Furthermore, none of those trials demonstrated any effect on the time to clinical worsening. The fact that the four-year survival rate of the patients who are not operated is quite independent of whether or not they receive specific PH drugs [37] could be attributed to their ineffectiveness in terms of effective mPAP reduction, although they target the microvascular disease and may increase the cardiac index.

For patients with surgically accessible disease, pulmonary endarterectomy (PEA) is the standard of care, as it is potentially curative. This surgical technique focuses on the mechanical component of the disease by removing obstructive, adherent chronic thromboembolic lesions from within the pulmonary vascular bed. Postoperative hemodynamics becomes normal or near normal in most patients after PEA. As shown from the University of California, San Diego (UCSD; San Diego, CA, USA) database including 1500 patients, mPAP can improve markedly following PEA (from 46 to 26 mmHg) [38]. A similar reduction in mPAP by PEA is demonstrated in another two registries with 314 and 880 patients, respectively [39,40]. The importance of lowering mPAP can also be supposed from the outcome of patients after endarterectomy. Persistent pulmonary hypertension after surgery remains the most important cause of early postoperative morbidity and mortality. In the international CTEPH registry, persistent pulmonary hypertension was associated with a higher early mortality [41], whereas in the UK national cohort [40], a higher mPAP, among other parameters, was negatively correlated with long-term survival in multivariate analyses. Of note, an mPAP ≥ 38 mmHg and a PVR ≥ 425 dyn·s·cm^−5^ identified those patients as being at a higher risk of death because of CTEPH. The reduction in afterload parameters also translates into a complete recovery of right and left ventricular geometry soon after PEA, and this right ventricular reverse remodeling is not limited by pre-existing ventricular size, shape, or function before correction [42]. These alterations in the structure and function of the RV are also reflected in the better survival of patients who undergo PEA in relation to those who do not undergo surgery [37].

**Figure 1:**
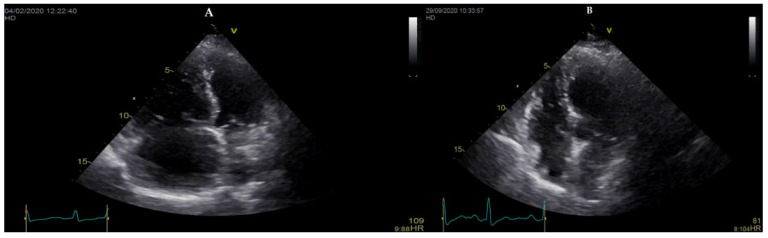
RV reverse remodeling after 7 sessions of BPA in an 80 years old woman with CTEPH and mPAP 63 mmHg at baseline (**A**) and 25 mmHg after the end of treatment (**B**).

Balloon pulmonary angioplasty (BPA) has become an established treatment for selected patients with inoperable CTEPH or persistent/recurrent PH after PEA, improving hemodynamics, right heart function, and exercise capacity [5], and like PEA, also focuses on the mechanical component of the disease. With BPA, mPAP can be reduced by 20%–>40% of the baseline values [43], more than double what can be achieved with specific drugs. Findings from a systematic review and meta-analysis indicate the greatest efficacy of BPA on hemodynamic and functional parameters compared to medical therapy; mPAP was reduced by a mean value of 14.8 mmHg, compared to only 4.9 mmHg with the PH drugs [44]. Additionally, if a meta-analysis carries several limitations, two randomized clinical trials compared the effectiveness of BPA with that of riociguat, which is the only oral-specific PH drug approved for the treatment of CTEPH, and both demonstrated the same substantial reduction in mPAP with BPA [45,46]. As right heart failure is also the leading cause of death in CTEPH, RV function could be considered the major determinant of prognosis. BPA improves hemodynamics, and this effect seems to result in reverse RV remodeling and improvement in RV function (Figure 1).

A systematic review confirmed the beneficial effect of BPA on several echocardiographic and cardiac MRI indices as the result of RV remodeling and in accordance with hemodynamic improvement [47]. In a meta-analysis including 299 patients, BPA resulted in obvious RV morphological changes and improvements in RV global systolic performance in the short term after the improvement of pulmonary hemodynamics, and especially mPAP and PVR; these findings were suggestive of RV reverse remodeling [48]. This reduction in afterload with BPA and subsequent reverse RV remodeling resulted in a significantly improved survival rate of up to 98.9% at 3 years [49]. Furthermore, the two invasive techniques for the treatment of CTEPH, with their effectiveness in reducing the afterload, clearly offer better survival to patients in relation to medication, as shown in the Inami et al. study, where the 5-year survival rate was 98%, vs. 64% in the drug group [50].

## 7. Conclusions

All these lessons from drug therapy or transplantation in PAH patients and the invasive treatment of CTEPH give us a common conclusion: the rigorous lowering of mPAP is feasible, and this should be the target of therapy. Functional parameters such as the 6MWD or WHO class are very important for assessing risk, but patients will still die if we do not focus on the RV. We now have a lot of proof of how and what can reverse the remodeling of the RV, and this should be the guide for changing our therapeutic strategy. Aggressive initial combination therapy including parenteral prostanoids under regular clinical and imaging follow-up should be the treatment strategy to enable right ventricle reverse remodeling, leading to remarkable hemodynamic amelioration. Moreover, pulmonary artery pressure-directed multi-drug therapies in PAH may reverse pulmonary vascular disease, so the near normalization or normalization of mPAP can be the target of therapy. Further studies are needed to validate mPAP as a primary endpoint in PAH drug trials, and new drugs targeting other pathophysiological pathways could be, in the future, more effective in contributing to a better survival rate.

## Figures and Tables

**Table 1:** Underlying treatment strategy including parenteral prostanoids, with a prominent effect on reduction in mPAP and on survival improvement of pulmonary arterial hypertension.

Study	Treatment	mPAP Reduction	Survival
Sitbon et al. 2014 [22]	i.v. epoprostenol + bosentan + sildenafil	32%	3 yr: 100%
Ogawa et al. 2017 [23]	i.v. epoprostenol + oral therapy	>37%	1 yr: 97.9%, 3 yr: 92.1%
Akagi et al. 2010 [24]	i.v. epoprostenol monotherapy	29%	3.7 yr: 100%
Ogawa et al. 2014 [25]	i.v. epoprostenol + oral therapy	44%	5 yr: 96% ^1^
Badagliacca et al. 2022 [26]	parenteral prostanoid + oral therapy	cut-off value of 35 mmHg	1 yr: 90%
D’Alto et al. 2020 [27]	s.c. Treprostinil + ambrisentan + tadalafil	30%	2 yr: 100%
Tokunaga et al. 2016 [28]	i.v. epoprostenol + oral therapy	38% ^2^	9.5 yr: 100% ^2^
Sugiyama et al. 2022 [30]	i.v. epoprostenol + oral therapy	<25 mmHg in 47% of pts	5 yr: 97.3%

^1^ In patients who achieved mPAP < 42.5 mmHg, the 10-year survival rate was 100%. ^2^ In rapid increase group.

## Data Availability

No new data were created or analyzed in this study. Data sharing is not applicable to this article.
